# Invasive marine species discovered on non–native kelp rafts in the warmest Antarctic island

**DOI:** 10.1038/s41598-020-58561-y

**Published:** 2020-01-31

**Authors:** Conxita Avila, Carlos Angulo-Preckler, Rafael P. Martín-Martín, Blanca Figuerola, Huw James Griffiths, Catherine Louise Waller

**Affiliations:** 1 Department of Evolutionary Biology, Ecology, and Environmental Sciences, University of Barcelona & Biodiversity Research Institute (IRBio), Av. Diagonal 643, 08028 Barcelona, Catalonia Spain; 2 Department of Biology, Healthcare and the Environment, University of Barcelona & Biodiversity Research Institute (IRBio), Av. Diagonal 643, 08028 Barcelona, Catalonia Spain; 3 Institute of Marine Sciences (ICM-CSIC), Pg. Marítim de la Barceloneta 37-49, 08003 Barcelona, Catalonia Spain; 4 British Antarctic Survey, High Cross, Madingley Road, Cambridge, CB30ET England; 5 University of Hull, Department of Biological and Marine Sciences, Cottingham Road, Hull, HU6 7RX UK

**Keywords:** Ecology, Environmental impact

## Abstract

Antarctic shallow coastal marine communities were long thought to be isolated from their nearest neighbours by hundreds of kilometres of deep ocean and the Antarctic Circumpolar Current. The discovery of non–native kelp washed up on Antarctic beaches led us to question the permeability of these barriers to species dispersal. According to the literature, over 70 million kelp rafts are afloat in the Southern Ocean at any one time. These living, floating islands can play host to a range of passenger species from both their original coastal location and those picked in the open ocean. Driven by winds, currents and storms towards the coast of the continent, these rafts are often cited as theoretical vectors for the introduction of new species into Antarctica and the sub-Antarctic islands. We found non-native kelps, with a wide range of “hitchhiking” passenger organisms, on an Antarctic beach inside the flooded caldera of an active volcanic island. This is the first evidence of non-native species reaching the Antarctic continent alive on kelp rafts. One passenger species, the bryozoan *Membranipora membranacea*, is found to be an invasive and ecologically harmful species in some cold-water regions, and this is its first record from Antarctica. The caldera of Deception Island provides considerably milder conditions than the frigid surrounding waters and it could be an ideal location for newly introduced species to become established. These findings may help to explain many of the biogeographic patterns and connections we currently see in the Southern Ocean. However, with the impacts of climate change in the region we may see an increase in the range and number of organisms capable of surviving both the long journey and becoming successfully established.

## Introduction

Human activity and shipping have long been considered the principal threats to the “biosecurity” of the remote and isolated shallow marine ecosystems of Antarctica^1^. However, recent work has shown that the Southern Ocean’s (SO) strong, circumpolar winds, currents and fronts may not be a barrier to natural colonization from the north^2–4^. Floating kelp is a potential vector for distributing species across the vast oceanic distances between the sub-Antarctic islands. It has been estimated that there may be over 70 million kelp rafts afloat at any one time in the Sub-Antarctic, 94% of which are *Durvillaea antarctica*^5^. The remote archipelagos distributed between 45 and 60° S are key locations for dispersal either side of the Polar Front (PF) and across^2–4,6^. The discovery of the non-Antarctic bull kelp, *D. antarctica* on Antarctic beaches, coupled with oceanographic models, demonstrate a non-anthropogenic mechanism for species introduction into Antarctica^4^. Genomic analyses revealed that the kelp specimens originated in the sub-Antarctic (Kerguelen Island and South Georgia) and dispersed thousands of kilometres to reach the Antarctic coast^4^. The only epibionts found on these specimens were goose barnacles (*Lepas australis*), and this epipelagic species is likely to have colonised the kelp during its time drifting in the open ocean^4^.

Deception Island (DI) is an active volcano in the South Shetland Islands, located off the West Antarctic Peninsula. The flooded caldera of DI is species poor in comparison with neighbouring islands due to recent eruptions (1970) and ongoing volcanic activity^7^. Recent work shows an increasing biodiversity gradient towards the entrance of the bay^7,8^. The geothermal and morphological nature of the caldera provides a relatively calm and warm-water habitat, with bottom water temperatures of about 2–3 °C, protected from ice disturbances (ice scouring, anchor ice, etc), perhaps offering favourable habitat for potential invasive species entering Antarctica.

Macroalgal rafting has been suggested to explain similarities in species composition and low genetic differentiation of intertidal marine communities across the sub-Antarctic^9–11^. This hypothesis implies some degree of successful colonization or mixing of the transported species with native sub-Antarctic species. However, all the possible natural pathways at both sides and across the PF result in a low probability that an individual raft will ever make landfall at a site with suitable characteristics for colonisation, given the vastness of the SO and the small size of most of the islands^12^. If a species succeeds to establish a local population, however, it may face little competition for resources and space, and may thrive^13^. In this context, thus, DI could represent a proxy for what may happen in other parts of Antarctica.

Marine species may reach Antarctic waters by a number of different dispersal mechanisms. Rafting on floating macroalgae is likely to be the biggest vector for natural dispersal into Antarctic waters. In a similar passive way, plastics have also been reported to carry a variety of epibionts in Antarctic waters^14^. Bryozoans are effective colonizers of surfaces and one of the most important components of biofouling assemblages^15,16^. Five bryozoan species were found attached to a plastic debris collected on Adelaide Island (Antarctic Peninsula)^14^. All of these species were endemic to the Antarctic and it was estimated that debris had been in the water for at least 1 yr. Most colonies were reproductively active, having the possibility of releasing larvae during transportation. In fact, the cyphonaute larvae of the bryozoan *M. membranacea* have been found in ballast water^17^, and their colonies can raft on kelp, such as *Macrocystis* spp and *Nereocystis* spp, as well as on plastic debris^18^. Fraser *et al*.^19^ reported 10 invertebrate species rafting on algae for at least 400 km, during several weeks, between New Zealand and the neighbouring sub-Antarctic islands.

The recent discovery of fresh specimens of the non-native giant kelps (*Macrocystis pyrifera* and *D. antarctica*) with a range of epibiotic animals and algae as passengers, washed up on the shores of Deception and Livingston Islands, provides a unique opportunity to study a potential colonisation event. Here we present the first evidence of non-native shallow water epibiotic organisms reaching Antarctica by long-term rafting. By identifying the species found living on the kelp and examining their distributions we assess the potential impacts of these species becoming established.

## Methods

**Figure 1:**
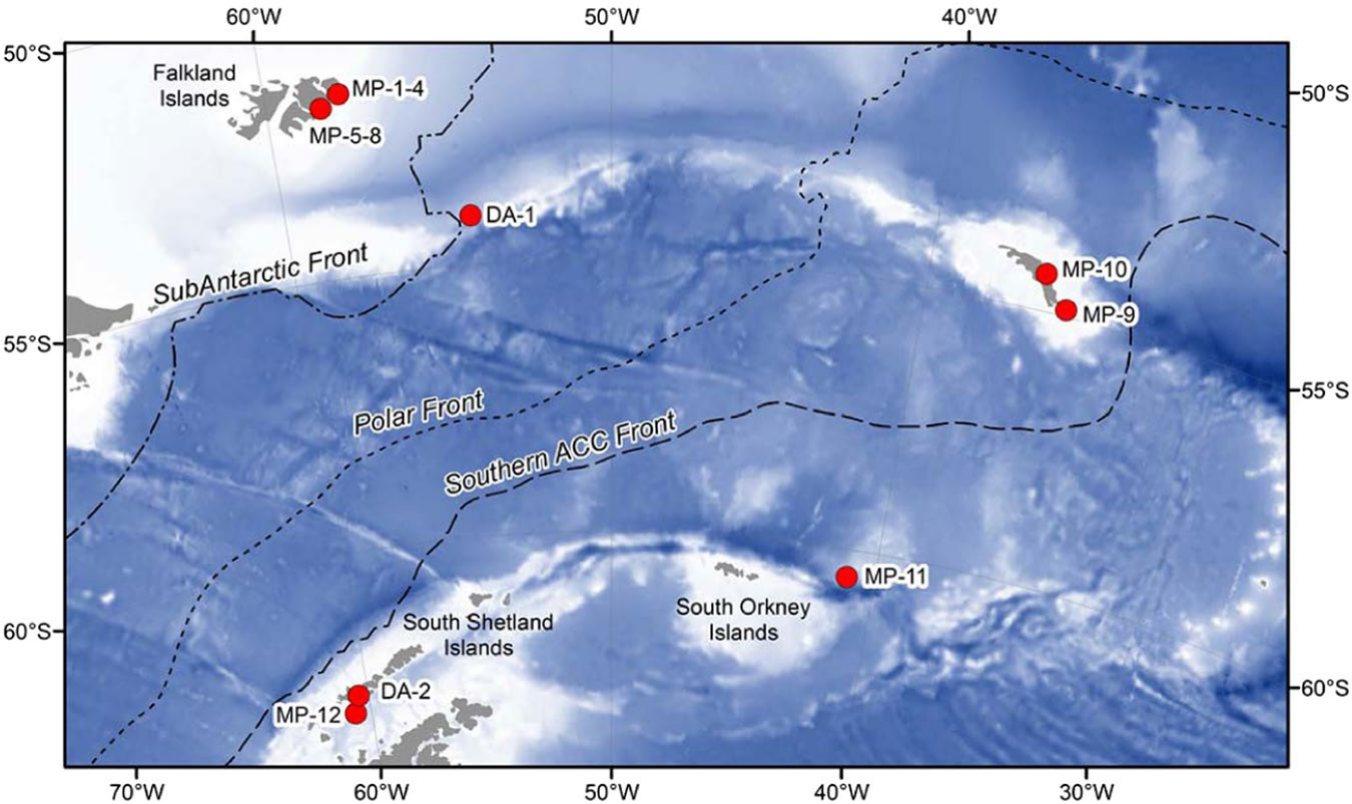
Map of the collecting localities showing the Polar Front (dotted line) and sampling points (in red). DA (*Durvillaea antarctica*), MP (*Macrocystis pyrifera*). MP-1–4: Falkland Islands (North), MP-5-8: Mare Harbour (Falkland Islands), DA-1: South of Falkland Islands (Drake passage), MP-9: South Georgia Islands (South), MP-10: South Georgia Islands (North), MP-11: South Sandwich Islands, and MP-12: Deception Island (South Shetland Islands), DA-2: Livingston Island (South Shetland Islands).

Samples were collected from the sub-Antarctic to Antarctic islands (Fig. 1, Table 1). Twelve rafting floating kelps were collected on both sides of the PF during the Antarctic expedition of the RRS James Clark Ross in 2016. Two more kelps were collected South of the PF. *M. pyrifera* was collected on the beach in DI (South Shetland Islands) during the Distantcom-2 Antarctic cruise in February, 2017. *D. antarctica* fragments were collected on the beach in front of the Spanish station in Livingston Island in February, 2019 during the Bluebio-2 cruise. Samples were photographed and frozen for further identification of the seaweeds and their epibionts. Samples of rafting kelp ranged from 0.5 to 18.1 kg wet weight. The passenger species traveling upon the kelps reached a total of 7534 specimens (538 ± 637 passengers/kelp, within a range from 0 to 2362 per kelp) and were identified to the lowest possible taxonomic level in the laboratory. The entire rafts were sampled for fauna. Identification of seaweed samples was achieved by studying morphological features, as well as histological examination of the thallus.

**Table 1:** Rafting kelp collected in this study.

Code	Species	Place	Lat (S)	Lon (W)	Polar Front	Date (mm/yy)
MP-1	*Macrocystis pyrifera*	Falkland Islands	−51,690	−57,865	North	02/16
MP-2	*Macrocystis pyrifera*	Falkland Islands	−51,690	−57,865	North	02/16
MP-3	*Macrocystis pyrifera*	Falkland Islands	−51,690	−57,865	North	02/16
MP-4	*Macrocystis pyrifera*	Falkland Islands	−51,690	−57,865	North	02/16
MP-5	*Macrocystis pyrifera*	Mare Harbour (Falklank Is.)	−51,903	−58,423	North	03/16
MP-6	*Macrocystis pyrifera*	Mare Harbour (Falklank Is.)	−51,903	−58,423	North	03/16
MP-7	*Macrocystis pyrifera*	Mare Harbour (Falklank Is.)	−51,903	−58,423	North	03/16
MP-8	*Macrocystis pyrifera*	Mare Harbour (Falklank Is.)	−51,903	−58,423	North	03/16
DA-1	*Durvillaea antarctica*	South Falkland Islands	−54,110	−54,340	North	02/16
MP-9	*Macrocystis pyrifera*	South Georgia	−54,880	−35,514	South	03/16
MP-10	*Macrocystis pyrifera*	South Georgia	−54,326	−36,382	South	03/16
MP-11	*Macrocystis pyrifera*	South Sandwich Islands	−60,52	−41,04	South	03/16
MP-12	*Macrocystis pyrifera*	Deception Island	−62,9789	−60,657	South	02/17
DA-2	*Durvillaea antarctica*	Livingston Island	−62,661	−60,398	South	02/19

MP: *Macrocystis pyrifera*. DA: *Durvillaea antarctica*.

### Passengers into the cold

**Figure 2:**
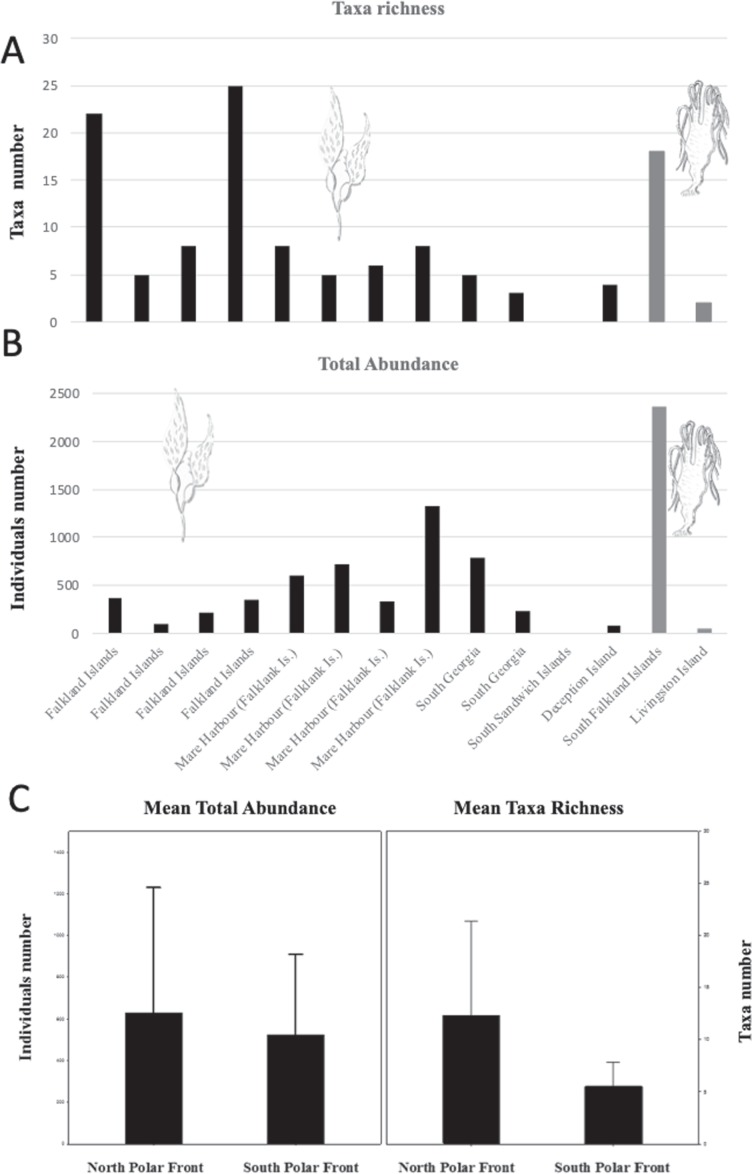
Abundance and taxa richness of epibionts in the rafting algae studied here. Taxa richness (**A**). Total abundance (**B**). Black bars: *Macrocystis pyrifera*, Grey bars: *Durvillaea antarctica*. Means of total abundance and taxa richness (**C**) at North and South of the Polar Front (PF) (± s.d.).

**Figure 3:**
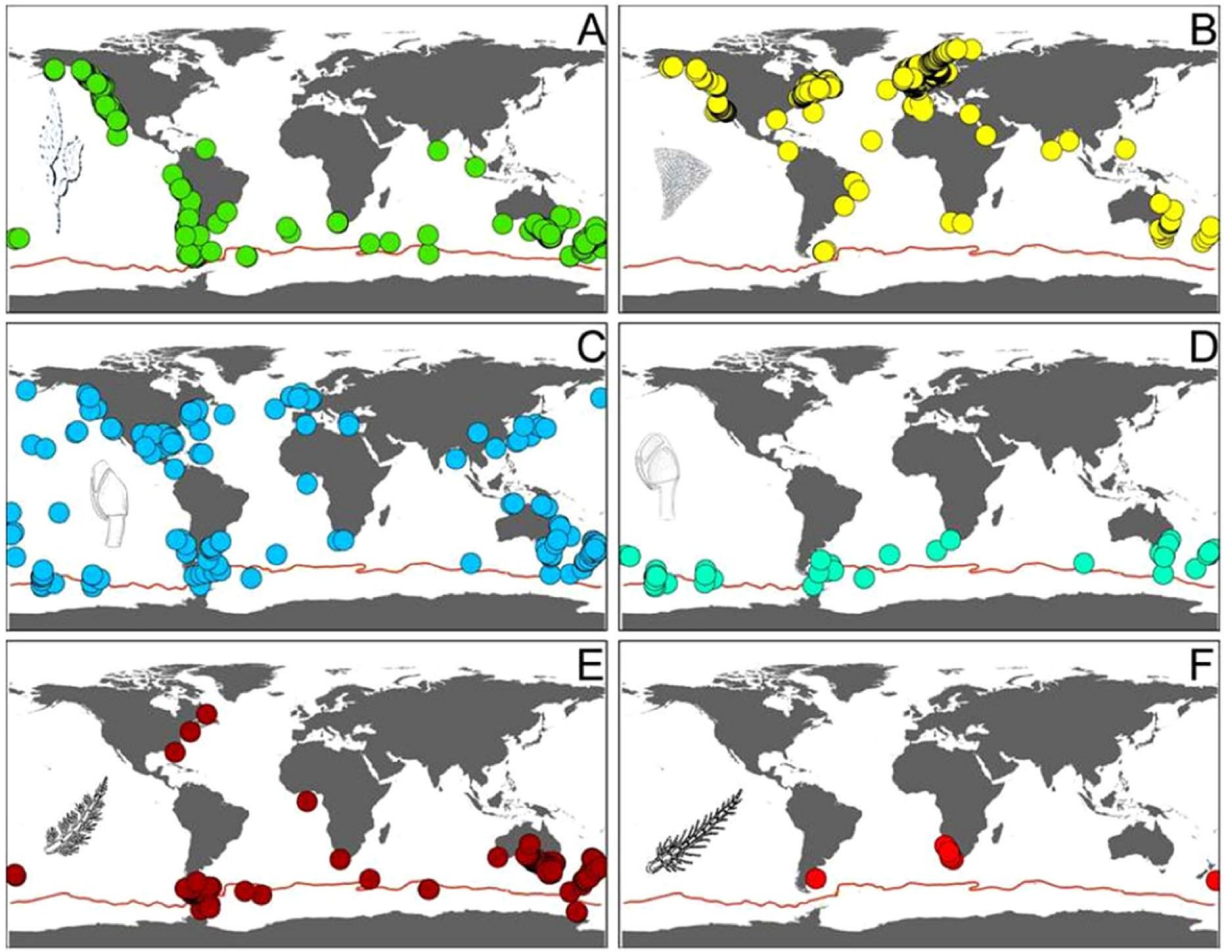
Known distributions of the epibiotic species found associated with *Macrocystis pyrifera* (**A**: Distribution of *M. pyrifera*) on the South Shetland Islands: *Membranipora membranacea* (**B**); *Lepas anatifera* (**C**); *Lepas australis* (**D**); *Ballia callitricha* (**E**) and *Ballia sertularioides* (**F**). Data from GBIF^45^.

Abundance and taxa richness of epibionts found on floating macroalgae in the Southern Ocean vary between the species of kelp *(M. pyrifera* and *D. antarctica*) and the individual rafts (Fig. 2). Other rafts, including those formed by *D. antarctica*, were observed at DI but were not sampled for fauna. Among the four passenger species found alive on *M. pyrifera* in DI, the most significant in terms of potential ecological impact, other than the non-native kelp itself, is the cheilostome bryozoan *Membranipora membranacea*. This is a well-known encrusting species with a proven ability to colonise new environments and cause significant damage to ecosystems by limiting the ability of seaweeds to reproduce and grow^20^. This bryozoan is widely distributed in temperate oceans with distinct populations in the Pacific (North Pacific, Chile, Australia and New Zealand) and Atlantic oceans (North East Atlantic and South Africa) (Fig. 3). *M. membranacea* has become an established invasive species in the North West Atlantic along the coast of North America and has caused extensive losses of kelp canopy through a process of defoliation^21^. Although the species is recorded as far north as northern Scandinavia in the Arctic, it has never been previously reported from south of the PF, but it is likely to already be well adapted to cold water conditions, therefore posing more than a hypothetical risk for Antarctic waters.

The combination of having a long-lived planktonic larva (from 2 weeks to 2 months), sexual (hermaphroditic zooids) and asexual reproduction, fast growth rates, effective food acquisition in a wide range of flow rates, ability to form large colonies and to colonize kelps make *M. membranacea* a successful disperser, colonizer, and invasive species^22–24^. Potentially, these kelp can be transported much farther than bryozoan larvae^25–31^. Furthermore, their heavy encrustations may have a negative impact on marine ecosystems by increasing the brittleness of kelp blades, followed by extensive losses of kelp canopy^21^, and by limiting the ability of the seaweeds to reproduce and grow, specifically interfering with spore release from the kelp blade^20^. It has also been shown that other species of the same genus may block nutrient uptake and photosynthesis^32,33^.

The other three species found alive on the kelp in DI have all been previously reported south of the PF. *Ballia callitricha* and *B. sertularioides* are shallow water red algae with a general Southern Hemisphere distribution that includes previous records from the Ross Sea, Antarctica (Fig. 3), but not from DI or West Antarctica^8^. Juveniles and adults of the southern goose barnacle *Lepas australis*, were also found. This species, commonly found attached to floating substrata such as macroalgae, volcanic pumice, and plastics in the Southern Ocean (Fig. 3), was the only species recently reported on a specimen of giant bull kelp, *D. antarctica*, found on King George Island, also part to the South Shetland Islands group^4^.

Using growth rates cited by Fraser *et al*.^4,19^ we estimate an age of approximately 30 days for the barnacles*, L. australis*, found at DI, suggesting that colonization happened in the open sea. Alternative, faster transportation mechanisms may also exist (e.g. shipping vectors and heavy storms). In fact, Lewis *et al*.^34^ suggested hull-fouling is likely to be the most important vector for transporting species to Antarctica as ships create novel pathways, moving across currents and often visiting many locations over short periods of time. The increasing ship activity appears to be a very important factor increasing the probability of non–native marine species establishing within the Antarctic region in the coming decades (over 180 ships were active around Antarctica and the sub‐Antarctic islands in 2017–2018, on potentially more than 500 voyages)^35^. The presence of small–sized specimens of *L. anatifera* in the kelp found at Livingston Island could also indicate a short-term rafting for this species. Abundant, alive *L. anatifera* specimens found on *D. antarctica* fragments in South bay, Livingston Island, represent, in fact, the first Antarctic report for the species, which was described in tropical and subtropical waters of South America^36^. The potential effects of barnacle colonization in Antarctica are unknown, but in fact, being pelagic rafting species, they seem unlikely to pose any real threat to the shallow water ecosystems, especially as *L. australis* is already commonly found on rafts and litter in the Southern Ocean. However, their heavy growth could sink the kelp, thus facilitating access to the seafloor for other benthic passengers.

The other floating and beached kelp samples (*M. pyrifera* and *D. antarctica*) collected from either side of the PF were found to be carrying organisms within 12 different phyla as passengers (Tables 1 and 2). Each kelp raft examined represented a different, although sometimes overlapping, subset of organisms usually found inhabiting shallow marine habitats. Only one of the floating specimens, MP-11, an example of the non-Antarctic *M. pyrifera* found near the South Orkney Islands, had no passengers at all. The most commonly found taxa included amphipod crustaceans, polychaete worms, molluscs, and bryozoans (Table 2). The DI floating kelp was the only specimen collected south of the PF carrying *M. membranacea*, although this bryozoan was frequently found at the Falkland Islands, a potential source of kelp rafts in that region^3^. Although more studies are needed to know if *M. membranacea* has become established in the SO, the potential for this species to impact Antarctic ecosystems could be high, not only in DI, as macroalgal substrates are widespread and colder temperatures are not preventing its spread. For example, a recent study based on a baseline data on presence/absence and abundance of this bryozoan near its current northern range limit suggests that the available algal substrate may be more important than temperature in limiting the spread and abundance of *M. membranacea*^37^. On the other hand, MP-5, collected from the open ocean north of the PF, was heavily encrusted with thousands of adult and juvenile goose barnacles. This specimen was also host to a rich and varied community of other organisms that are likely to have been associated with the raft before it became dislodged (Table 2).

**Table 2:** Organisms found as passengers on the kelp raft in this study (numbers indicate counts).

Phyllum	Taxa	MP-1	MP-2	MP-3	MP-4	MP-5	MP-6	MP-7	MP-8	DA-1	MP-9	MP-10	MP-11	MP-12	DA-2
Rhodophyta	*Ballia callitricha*													1	
Rhodophyta	*Ballia sertularioides*													1	
Porifera	Porifera				2	2									
Cnidaria	Anthozoa					3									
Cnidaria	Hydrozoa	1				13				3		5	7		
Bryozoa	Bryozoa	1	1	1	20	18	5	3		17	27	5	120		
Bryozoa	Cyclostomatidae			2											
Bryozoa	*Membranipora membranacea*													1	
Entoprocta	Entoprocta						100								
Mollusca	Mollusca				1										
Mollusca	Bivalvia			4	2		500			27	71	3	39		
Mollusca	Gastropoda				2					2					
Mollusca	*Kidderia* sp.					2									
Mollusca	*Scurria scurra*					1									
Mollusca	*Gaimardia trapesina*	97					76	30							
Mollusca	Nudibranchia						2								
Mollusca	*Nacella*	2													
Mollusca	*Nacella mytilina*	2													
Mollusca	Fissurellidae				1										
Platyhelminthes	Platyhelminthes				1	1									
Annelida	Nemertea									1			2		
Annelida	Polychaeta									3	601	5	1005		
Annelida	Polynoidae	1			8	1									
Annelida	Cirratulidae				2										
Annelida	Serpulidae	101	100	100	105		100	200							
Annelida	Syllidae	6			2										
Annelida	Terebellidae	7			8										
Annelida	Nereidae	3			4										
Annelida	Capitellidae	5													
Annelida	*Torodrilus* sp.														1
Annelida	Sabellidae	1													
Sipuncula	Sipuncula				3										
Arthropoda	Insecta	1													
Arthropoda	Haplocheira	78													
Arthropoda	Harpacticoida	40													
Arthropoda	Calanoida	1													
Arthropoda	Pedunculata					2230									
Arthropoda	*Joeropsis curvicornis*					1									
Arthropoda	Caprellidae					1									
Arthropoda	Pantopoda					1									
Arthropoda	Ostracoda					25									
Arthropoda	Cucumariidae				15										
Arthropoda	Eusiridae			13	25	1									
Arthropoda	Isopoda									56	10	31	33		
Arthropoda	Munnidae					1									
Arthropoda	Amphipoda		2	50	42	55				493	20	280	115		
Arthropoda	Corophiidae		2	50	65										
Arthropoda	Ischyroceridae				3	5									
Arthropoda	*Ischyromene eatoni*	1													
Arthropoda	*Halicarcinus planatus*	5			19										
Arthropoda	*Plakarthrium punctatissimum*	1													
Arthropoda	*Peltariom spinulosum*	1													
Arthropoda	*Exosphaeroma lanceolatum*	1													
Arthropoda	*Lepas australis*													76	
Arthropoda	*Lepas anatifera*														50
Echinodermata	Echinoidea				3										
Echinodermata	Asteroidea				9										
Echinodermata	Ophiuroidea				3										
Echinodermata	Apodida				1										
Chordata	Actinopteri					1									
	Seaweed	4	1	1	2							5	5		

MP: *Macrocystis pyrifera*. DA: *Durvillaea antarctica*.

Other significant findings included the brachyuran crabs *Halicarcinus planatus* and *Peltariom spinulosum* in the *M. pyrifera* fragments washed up on the shore at the Falkland Islands. *H. planatus* was first recorded in Antarctica at the shores of the South Orkney Islands in 1903^38^. It was reported again by Aronson *et al*.^39^ at the external side of the caldera of DI, supporting the hypothesis that DI could be the entrance gate for non-native species. *H. planatus* is a widely-distributed species in temperate waters, found from New Zealand to the Falklands and southern South America, as far north as Peru and Argentina^40,41^. *H. planatus* has also been found alive on floating kelp^42^. Although *H. planatus* was not found in our previous studies at Deception and Livingston Islands^8^, we did find it on *M. pyrifera* washed up on the shore of the Falkland Islands (pers data 2016, SO-AntEco expedition), which could easily be re-floated by high tides or rough weather. The impact of these crabs on local species is not known but could potentially be devastating due to the absence of durophagous fauna in Antarctic shallow benthic ecosystems^43,44^.

### Rafting to the south

**Figure 4:**
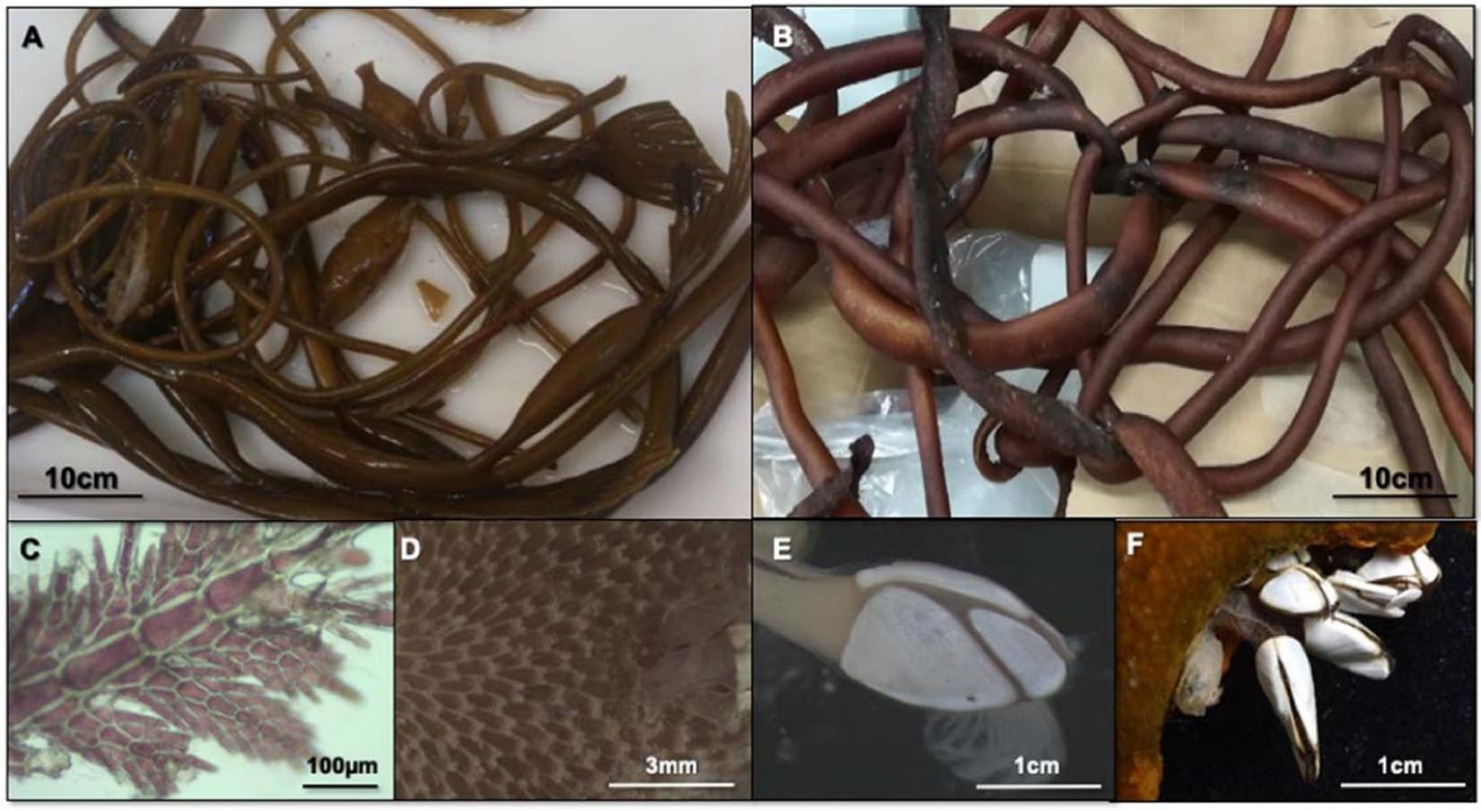
Rafting kelp and passengers. *Macrocystis pyrifera* (**A**) with passengers found at Deception Island, and *Durvillaea antarctica* (**B**) with cirripeda from Livingston Island, South Shetland Islands, Antarctica. (**C**–**F**) Passengers found on *M. pyrifera* at DI: the red alga *Ballia callitricha* (**C**); the bryozoan *Membranipora membranacea* (**D**); the cirripeda *Lepas australis* (**E**); and the cirripeda *L. anatifera* (**F**) on *D. antarctica* from Livingston Island.

The transport of organisms on ships’ hulls or in ballast water can take less than 4% of the time it would take to reach the same destination by rafting^11^. Although this significant reduction in time taken to reach Antarctica might allow a wider range of species to reach the continent alive, they would still need to be capable of surviving the conditions at their destination in order to become established. As such, our observation of a species with a documented track record of invasive and negative ecological impacts, such as *Membranipora membranacea*, in an active volcano (DI), with warmer, more favourable conditions, is very significant. The species reported here are common and well-distributed organisms and thus have the potential to persist or even thrive in the milder conditions of the caldera of DI (Fig. 4). It could only be a matter of time before some of these species acclimatize to the Antarctic environment and spread. These findings are even more relevant in the current context of global change, which could facilitate the survival of these species in other Antarctic environments once settled in favourable areas, such as DI, further reaching other places around the Antarctic peninsula. Therefore, these species may be useful indicators of climate change in Antarctic habitats and should be carefully monitored during the next years.

## Conclusions

Non-native, non-Antarctic kelp is reaching Antarctica now and again, particularly at Deception and Livingston Islands. DI is a key location for first colonisation of Antarctica due to its strategic location and the higher temperature of seawater compared to adjacent areas. The presence of passengers on the kelp, especially *Membranipora membranacea* and *Lepas anatifera* (as well as *Halicarcinus planatus* in the water outside the DI caldera) demonstrate that natural colonisation, or invasion, can happen at any time. Actually, *M. membranacea* has already become an invasive species in many places outside of Antarctica, and it is believed to have a potentially negative impact on marine ecosystems. Effects of passengers in Antarctic ecosystems are largely unknown, and therefore, we believe that monitoring these potentially invasive species in the frame of global change is crucial in the coming years.
